# Short review of biparametric prostate MRI

**DOI:** 10.1007/s12254-018-0458-1

**Published:** 2018-11-12

**Authors:** Fabian Steinkohl, Renate Pichler, Daniel Junker

**Affiliations:** 1 Department für Radiologie, Medizinische Universität Innsbruck, Anichstr. 35, 6020 Innsbruck, Austria; 2 Universitätsklinik für Urologie, Medizinische Universität Innsbruck, Anichstr. 35, Innsbruck, 6020 Austria; 3 Diagnostische und Interventionelle Radiologie, Landeskrankenhaus Hall in Tirol, Milser Str. 10, Hall in Tirol, 6060 Austria

**Keywords:** Prostatic neoplasms, Magnetic resonance imaging, Diagnostic imaging, Sensitivity and specificity, Screening

## Abstract

Magnetic resonance imaging (MRI) of the prostate has become the gold standard for visualization of prostate cancer. Prostate MRI is usually performed as multiparametric MRI (mpMRI). Since mpMRI has several drawbacks, a biparametric MRI (bpMRI) of the prostate has been proposed. Many studies have been published on mpMRI and bpMRI in recent years. This short review offers an overview of the latest developments in this rapidly evolving field of research.

## Introduction

Prostate carcinoma (PCa) is the most common cancer in men [1]. PCa is suspected because of a positive digital rectal examination of the prostate or because of high values of prostate-specific antigen. This antigen is produced by normal but also by malignant prostatic epithelium [2] and is widely used as a screening tool for PCa. Usually, a systematic biopsy of the prostate is performed to clarify this suspicion. This approach has a sensitivity of only 50% [3]. If the systematic biopsy of the prostate is negative but PCa is still suspected, magnetic resonance imaging (MRI) of the prostate can be performed. MRI can identify suspicious lesions with a high diagnostic accuracy [4]. The first study on the diagnostic value of pelvic MRI was published in 1983 [5]. According to Hricak et al., it was unclear whether MRI can distinguish neoplastic nodules from benign prostatitis. The field of prostate MRI has been developing rapidly since then. A milestone was the introduction of the Prostate Imaging Reporting and Data System (PI-RADS) in 2012 [6]. PI-RADS standardized prostate MRI protocols and standardized image interpretation and reporting of prostate MRI. PI-RADS is also used to communicate imaging findings between radiologists and the referring physician. PI-RADS assigns scores for lesions in the prostate from 1 to 5. PI-RADS 1 means “clinically significant PCa is highly unlikely,” PI-RADS 5 means “clinically significant cancer is highly likely” (Table 1).

**Table 1:** PI-RADS scores and their definition according to the ESUR panel

PI-RADS score	Definition of the ESUR panel
1	Clinically significant cancer is highly unlikely
2	Clinically significant cancer is unlikely
3	Presence of clinically significant cancer is equivocal
4	Clinically significant cancer is likely
5	Clinically significant cancer is highly likely

*PI-RADS* Prostate Imaging Reporting and Data System, *ESUR* European Society of Urogenital Radiology

**Fig. 1:**
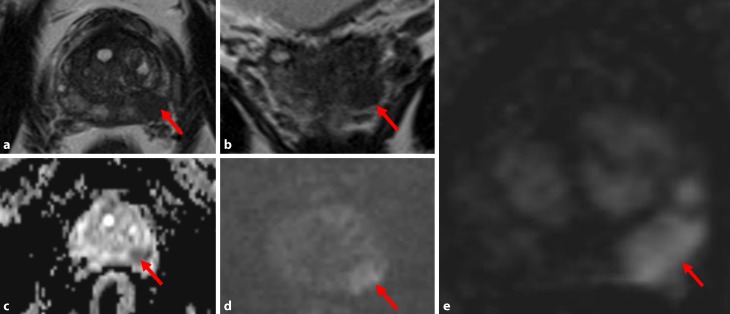
Prostate carcinoma in the peripheral zone (*red arrow*): hypointensity on axial T2-weighted (**a**) and on follow-up T2-weighted images (**b**); hypointensity on ADC (Apparent Diffusion Coefficient) maps (**c**) and hyperintensity on diffusion-weighted images (**d**); marked early contrast enhancement on dynamic contrast-enhanced sequences (**e**)

In recent years, prostate MRI is increasingly used to visualize PCa [7]. A new, simplified version, PI-RADS v2 was introduced in 2012 [8]. PI-RADS v2, too, is based on multiplanar T2-weighted sequences (T2w), diffusion-weighted sequences (DWI), and dynamic contrast-enhanced sequences (DCE). The prostatic anatomy is evaluated in the T2w sequences. The prostatic zones can be discriminated and important extraprostatic structures, such as the neurovascular bundles, can be seen. DWI is the key sequence in the PI-RADS system. PCa has a higher cell density than the surrounding normal prostatic tissue. Hence, the Brownian motion within the tumor is limited. This diffusion restriction can be visualized with DWI, but the spatial resolution of DWI is low. Intravenous contrast agent, usually gadolinium, has to be administered for the DCE sequences. DCE shows the contrast enhancement of the prostate over the time. PCa is believed to have an early gadolinium uptake and an early wash-out of the contrast agent. In the currently used PI-RADS v2, DCE plays a minor role. An early contrast enhancement in the DCE can lead to an upgrading from PI-RADS 3 to PI-RADS 4. Combining the aforementioned different sequences in one MRI examination has become known as multiparametric MRI (mpMRI; Fig. 1). MpMRI is well evaluated, but it has several drawbacks. To overcome some of them, a number of groups have proposed a “biparametric prostate MRI” (bpMRI).

This short review summarizes the latest findings on bpMRI for radiologists and referring physicians.

## Benefits of omitting DCE

In contrast to multiparametric MRI protocols, biparametric MRI protocols do not include DCE. Therefore, it has three major advantages: examination times are shorter, costs are lower, and the risk of adverse events with contrast agents is eliminated.

### Examination time

Omitting an MRI sequence shortens the examination time. There are wide differences in the literature on how much time can be saved by using a biparametric approach. Obmann et al. found 11.9 min scanning time (and 15.7 min table time) for bpMRI and ±45 min for mpMRI [9]. This was confirmed by Dong Hoon Lee et al., who found an in-bore time of 15 min for bpMRI and 45 min for mpMRI [10]. Other authors found considerably shorter time differences between mpMRI and bpMRI. Junker et al. reported a time reduction of 12 min using bpMRI [11] and another group found a time reduction of only 2.30 min [12]. A very fast biparametric MRI protocol that takes only 8.45 min has been published by Kuhl et al. [13].

### Costs for mpMRI

Performing a DCE involves direct costs for contrast agents and peripheral venous catheters as well as indirect costs for personnel and longer scanning times. Data for costs diverge in the literature. Junker et al. reported additional costs of about € 56 for a 70-kg patient only for the contrast agent [11]. A Korean group calculated costs of about $ 600 for mpMRI and only $ 300 for bmMRI [10].

### Risk associated with contrast medium

The contrast medium used for DCE in mpMRI is gadolinium-based. The risk of immediate hypersensitivity reactions to a gadolinium-based contrast medium is low [14]. Until recently, gadolinium was considered to be safe [15], but it has emerged recently that gadolinium can form depositions in the brain [16]. Therefore, caution is called for when gadolinium is administered [17]. In view of these findings, it seems to be advisable to reconsider the necessity of DCE.

## Diagnostic performance of mpMRI and bpMRI

Although DCE is part of the PI-RADS v2 guidelines [8] and is considered a cornerstone of prostatic MRI by some authors [18], its role is controversial in the literature. Greer and colleagues found that DCE significantly improves PCa detection in the peripheral zone of the prostate [19]. Some older studies found an improvement of PCa detection due to the use of DCE [20, 21]. A limited incremental value of 3% was calculated for DCE [22]. Recent studies state that DCE has no or only a limited role in PCa detection [23–27]. The suspicion was raised that DCE could potentially increase the number of false-positive examinations [13]. These findings resonate well in the scientific community. To date, 16 articles can be found on PubMed.gov using the search terms “biparametric prostate MRI” from January 2018.

Two large meta-analyses have been published this year. One analyzed 33 studies from the period 2007–2017 [28], another one analyzed 20 studies, all published before December 2017 [29]. Xiang-ke Niu and colleagues found a pooled sensitivity of 0.81 and a specificity of 0.77 for bpMRI for PCa detection [28]. Woo et al. reported a pooled sensitivity of 0.74 and a specificity of 0.90 for bpMRI and a pooled sensitivity of 0.76 and a specificity of 0.89 for mpMRI. Therefore, the authors conclude that bpMRI has the same diagnostic performance as mpMRI for the detection of PCa [29]. Recent single-center studies from 2018 published similar results [7, 9, 11, 12, 30].

## Reasons for using DCE

Although there are many good reasons for omitting the use of gadolinium-based contrast agents for prostate MRI, there are still situations in which DCE can be useful. DCE can be used to detect certain small PCa [24]. It can be used to monitor response to therapy after radical prostatectomy [31]. Additionally, we find that DCE helps to avoid typical pitfalls (e. g., in the anterior fibromuscular stroma) and to diagnose seminal vesicle infiltration or extracapsular extension.

## Future developments of prostate MRI

Current multiparametric MRI protocols are designed to answer a variety of clinical questions (see above; [13]). The demand for prostate MRI rises since prostate MRI is used as a PCa screening tool prior to biopsy. In view of the latest publications it seems feasible to use bpMRI in these cases. Scialpi and colleagues proposed a new and simpler version of PI-RADS [24] that is based on a bpMRI. In their simplified PI-RADS, DWI is the dominant sequence in the peripheral and transition zone. Furthermore, the maximal size of a category 3 lesion is 0.5 cm^3^. Another advantage is that bpMRI images are easier to read. While a large Norwegian study found a poor inter-reader agreement for mpMRI [32], an Italian group reported that there is excellent agreement between different readers with different levels of experience for bpMRI [7].

PI-RADS v2 will continue to evolve. We believe that the use of DCE is not necessary in every patient, but the indication for administering gadolinium-based contrast agents will have to be considered individually depending on the clinical question.

## Conclusion

During the past months, several studies have been published on biparametric prostate MRI protocols. All of them underline the feasibility of shorter biparametric protocols for PCa detection. In light of these findings, we believe that the need for administering contrast agents for prostate MRI has to be considered individually for each patient depending on the clinical question.
